# Mindsets and Neural Mechanisms of Automatic Reactions to Negative Feedback in Mathematics in Elementary School Students

**DOI:** 10.3389/fpsyg.2021.635972

**Published:** 2021-08-06

**Authors:** Ita Puusepp, Tanja Linnavalli, Milla Huuskonen, Karoliina Kukkonen, Minna Huotilainen, Teija Kujala, Sonja Laine, Elina Kuusisto, Kirsi Tirri

**Affiliations:** ^1^Faculty of Educational Sciences, University of Helsinki, Helsinki, Finland; ^2^Cognitive Brain Research Unit, Faculty of Medicine, University of Helsinki, Helsinki, Finland; ^3^Department of Psychology and Logopedics, Faculty of Medicine, University of Helsinki, Helsinki, Finland; ^4^Viikki Normal School, University of Helsinki, Helsinki, Finland; ^5^Faculty of Education and Culture, Tampere University, Tampere, Finland

**Keywords:** mindsets, implicit theories, math ability, feedback error-related negativity, P300, feedback

## Abstract

Neuroscientific research regarding mindsets is so far scarce, especially among children. Moreover, even though research indicates the importance of domain specificity of mindsets, this has not yet been investigated in neuroscientific studies regarding implicit beliefs. The purpose of this study was to examine general intelligence and math ability mindsets and their relations to automatic reactions to negative feedback in mathematics in the Finnish elementary school context. For this, event-related potentials of 97 elementary school students were measured during the completion of an age-appropriate math task, where the participants received performance-relevant feedback throughout the task. Higher growth mindset was marginally associated with a larger P300 response and significantly associated with a smaller later peaking negative-going waveform. Moreover, with the domain-specific experimental setting, we found a higher growth mindset regarding math ability, but not general intelligence, to be associated with these brain responses elicited by negative feedback regarding errors in math. This suggests that it might be important to address domain-specific and even academic-domain-specific beliefs in addition to general mindsets in research and practice.

## Introduction

Mindsets are defined as implicit beliefs individuals hold about basic human abilities and attributes, such as intelligence or personality (Dweck, 2006). They exist on a spectrum from fixed mindsets, which refer to believing that specific human attributes are static and unchangeable, to growth mindsets, which refer to believing that these attributes are malleable and can be shaped and developed with effort. Mindsets can be understood as meaning systems, which have an organizing function when it comes to people making sense of the world, interpreting their experiences, and planning their behavior (Dweck et al., 1995).

These meaning-making systems develop in constant interaction with the perceived environment of the person. Furthermore, while research among children suggests that during elementary school years, mindsets might still be in the process of development as organizational frameworks, they are nonetheless already related to achievement-related cognition and behaviors in theoretically predictable ways in the second half of elementary school (Kinlaw and Kurtz-Costes, 2007). The role of mindsets has been widely investigated in the educational context as they were shown to be related to various motivational and behavioral variables, including the way students handle academic setbacks and challenges (Blackwell et al., 2007; Aditomo, 2015). Namely, people with a fixed mindset are more prone to interpret their setbacks by attributing them to the lack of a rather stable ability when compared to people with a growth mindset, who rather attribute setbacks to the lack of effort (Dweck et al., 1995; Dweck, 2006). These differences in the interpretation of events can then lead to differences in the subsequent ways of coping with setbacks and the students’ psychological wellbeing. Growth mindset has been linked to students’ higher resilience, psychological wellbeing, and school engagement, which seem to be at least partly explained by the enhanced resilience (Zeng et al., 2016). Thus, it can be inferred that a better understanding of these implicit beliefs could be used to support students in their learning with regard to not only their academic achievement, but also their psychological wellbeing.

Mindsets are conceptually domain specific (Dweck et al., 1995), and it has been suggested that even though there seems to be a certain generality across mindsets regarding different domains, the specific domains of implicit beliefs are still distinguishable (Schroder et al., 2016). The general factor and domain-specific facets of mindsets were also apparent regarding their relations to psychological outcomes. Namely, specific mindsets specifically predicted the variance of psychological symptoms in that same domain, yet general mindset still moderately predicted the variance of symptoms in specific domains (Schroder et al., 2016). While Schroder et al. (2016) focused on distinguishing domain-specific mindsets regarding mental health, the research has previously focused on differentiating broader domains, such as intelligence, personality, and morality (Hughes, 2015).

Regarding the domain of intelligence, most of the research done on mindsets has focused on general intelligence without differentiating between possible subdomains of implicit beliefs, such as academic-domain-specific mindsets. Yet, recent research has also examined academic domain specificity of mindsets and shown that these beliefs can be distinguished between different academic domains already among first graders and that at least starting from teenage years they relate differently to academic-domain-specific motivation and achievement (Gunderson et al., 2017). Academic-domain-specific mindsets seem to predict outcomes in that specific academic domain better than general intelligence beliefs or mindsets regarding another domain (Gunderson et al., 2017; Costa and Faria, 2018).

Recently, there has been a growing interest in neuroscientific research on mindsets in order to gain a better comprehension of the mechanisms with which they associate with different behavioral outcomes. The so far scarce research conducted in this field has shown that there are differences in the event-related brain potentials (ERPs) between adults with growth and fixed mindsets (Mangels et al., 2006; Moser et al., 2011). ERPs are time-locked fluctuations of voltage recorded with electroencephalogram (EEG) regarding a certain event, for example, the presentation of a stimulus or execution of a response, such as the press of a button (Woodman, 2010; Kappenman and Luck, 2011). ERPs have been used for decades in research regarding perception and attention (Woodman, 2010; Kappenman and Luck, 2011). State-of-the-art instruments are mobile, so that the recordings can be performed in various environments, such as schools. The method has great temporal accuracy, thus enabling the observation of voltage fluctuations elicited by unfolding neural processes with great precision. This makes it possible to test hypotheses regarding rapid processing of information, which would otherwise be unobservable with using only behavioral methods. The opportunity to inspect the neural processes associated with perception and cognition of setbacks, such as errors and negative feedback, has made the technique useful also for researchers investigating the underlying mechanisms of mindsets (Tirri and Kujala, 2016).

Most of the ERP studies done on mindsets have focused on examining error-related ERPs in speeded reaction time tasks (Moser et al., 2011; Schroder et al., 2014, 2017). More specifically, they have explored error-related negativity (ERN) and error positivity (Pe), which are associated with adaptive behavioral adjustments following errors. ERN is a negative deflection that is elicited when an error is made (Gehring et al., 2011). It is maximal at midline frontocentral scalp locations and peaks at around 100 ms after an erroneous button press. The ERN is assumed to reflect processes involved in the evaluation of the need for control and its implementation (Gehring et al., 2011). Another ERP that has been explored to be elicited by errors is Pe. Pe is a slow positive-going waveform observed to follow the ERN in case of erroneous responses in speeded reaction time tasks. Pe has a more diffuse scalp distribution than ERN, and its maximum amplitude has in general been observed between 200 and 400 ms post-response (Overbeek et al., 2005). Pe has also been observed as a waveform consisting of two positive deflections, which have been termed as early Pe and late Pe (van Veen and Carter, 2002; Moser et al., 2011; Schroder et al., 2014). Even though the functional significance of Pe is still poorly known, the available data seem to suggest that it is mainly associated with error-awareness and the motivational significance of the committed error (Overbeek et al., 2005). Furthermore, for a more comprehensive understanding of the elicited brain responses, exploration of behavioral adjustment and their associations with the ERPs are suggested (Schroder and Moser, 2014). The widely used and recommended behavioral measure to study post-error adjustment and its associations with ERPs is post-error accuracy (PEA), which refers to the accuracy of the trials following errors. Other regularly reported behavioral adjustment measures are reaction times (RTs), including post-error RTs in relation to post-correct RTs referred to as post-error slowing (PES), but this has been differently interpreted and depends on task-specific parameters and, thus, has not been considered as reliable as PEA concerning post-error adjustment (Schroder and Moser, 2014). Importantly, ERN and Pe responses have been shown to relate to adaptive behavioral adjustments following errors (Torpey et al., 2011).

Exploring ERN and Pe and their associations with mindsets has resulted in informative findings. Namely, Moser et al. (2011) found higher growth mindset regarding general intelligence to be associated with higher PEA on a speeded reaction time task and a larger early and late Pe amplitude. They also found Pe to be positively correlated with PEA with Pe mediating the relationship between mindset and post-error performance.

Schroder et al. (2014) observed the effect of experimentally induced mindsets on ERPs. Differently from Moser et al. (2011) though, they found no association between early Pe and mindsets and demonstrated that late Pe was more positive in the fixed mindset condition than in the growth condition. They found smaller late Pe to be associated with enhanced stimulus processing ERP responses. Thus, Schroder and colleagues suggested that individuals in the growth mindset condition having a smaller late Pe prioritized stimulus processing instead of response processing. Regarding post-error behavior, though, they found no significant relationships between either of the Pe responses and PEA.

The only study in this field that has been conducted on children, as far as we know, found a higher growth mindset regarding general intelligence to be associated with a larger Pe difference between error and correct trials (Schroder et al., 2017). They also found that the relationship between mindset and PEA differed significantly between children with large versus small Pe difference amplitudes. Namely, growth mindset was associated with higher PEA in children with small Pe amplitudes, but not in children with large Pe amplitudes.

None of the previously mentioned studies have found mindsets to be associated with other post-error behavioral data than PEA, such as post-error reaction times (RTs) in speeded reaction time tasks. Neither have they found associations between mindsets and overall RTs or accuracy in the tasks used (Moser et al., 2011; Schroder et al., 2014, 2017).

Even though most of the ERP studies on mindsets have explored error-related brain responses, as far as we know, there is one study that focused on examining ERP responses elicited by feedback (Mangels et al., 2006). Indeed, negatively and positively displaced deflections have been observed to be elicited by performance-relevant feedback in addition to error commission. Namely, a negative deflection similar to ERN has been observed after presentation of feedback indicating incorrect performance, independent of the modality of the feedback (Miltner et al., 1997). Although this deflection has been observed to peak later than ERN, namely, between 200 and 350 ms after the onset of the feedback stimulus, it shares a similar scalp distribution (Miltner et al., 1997; Walsh and Anderson, 2012). As this ERP seems to result from cognitive processes associated with external feedback, it has been termed feedback-related negativity (FRN). Earlier research on error- and feedback-related ERPs has suggested that FRN appears to reflect the same neural process as ERN – a more generic neural process regarding initial detection of an outcome that is worse than expected (Miltner et al., 1997; Holroyd and Coles, 2002).

In addition to the negatively displaced FRN response, a positive-going waveform P300 has been found to be elicited by performance-relevant feedback. P300 response, which peaks approximately 300–600 ms after the eliciting stimulus, is not exclusive to negative feedback but is being generated when perceptual stimulus discrimination occurs and is thought to reflect the processing of attention-demanding stimulus more generally (Polich, 2007). It has initially been observed in oddball tasks, where it is elicited by infrequent target stimuli (Polich, 2007). P300 has later been suggested to be a canonical waveform, consisting of two subcomponents that reflect information processing: an earlier peaking P3a with maximum amplitude over frontal and central areas and a subsequent longer lasting P3b with a more parietal scalp distribution (Polich, 2007). P3a is sensitive to the novelty and rarity of the stimulus and is thought to index attention processes related to frontal working memory (Polich, 2007). It is sensitive to expectancy, with the response being the largest to unexpected stimuli (Butterfield and Mangels, 2003; Mangels et al., 2006; Polich, 2007). The subsequent longer lasting P3b subcomponent is thought to index memory processes (Polich, 2007). P300 seems to signal unexpected changes relevant for behavioral adjustment and has been assumed to reflect attentional processes, with larger amplitude associated with more and smaller amplitude less attentional resources being available for the processing of the stimulus (Polich, 2007). P300 amplitude has also been associated with learning from feedback. Namely, the amplitude of the feedback-locked P300 was shown to be larger for initial errors that were answered correctly in the subsequent retest when compared to initial errors that were not corrected in the retest (Butterfield and Mangels, 2003; Mangels et al., 2006; Ernst and Steinhauser, 2012). Interestingly, the positive-going ERP elicited after error commission – Pe – has been suggested to reflect similar neurocognitive processes to the ones reflected in P300. Namely, both Pe and P300 have been assumed to be involved in conscious processing of motivationally significant events (Ridderinkhof et al., 2009).

In the ERP study on mindsets that explored feedback-related brain responses, Mangels et al. (2006) used a general knowledge task and found differences in ERPs between growth- and fixed-minded participants. Namely, they observed differences regarding immediate performance feedback on the accuracy of the response and regarding learning-relevant feedback, which provided the correct answer to the previously presented question. Regarding performance feedback, fixed-minded participants had an enhanced anterior frontal P300 (peaking between 360 and 400 ms after the onset of the feedback stimulus) at Fz electrode site when compared to growth-minded participants. The authors suggested this to reflect fixed-minded participants’ heightened attention to performance feedback. Namely, they also found a larger anterior frontal P300 amplitude to be associated with endorsement of performance goals. Additionally, the results also indicated that a greater P300 amplitude at FCz was associated with higher error correction on the immediate subsequent retest. A greater P300 amplitude has been associated with better subsequent error correction in other studies as well (Butterfield and Mangels, 2003; Ernst and Steinhauser, 2012). The only FRN difference found between growth- and fixed-minded participants was a larger amplitude in the growth mindset group in case of expected errors. Regarding the behavioral measures, growth-minded participants performed better than fixed-minded participants on a surprise retest of initially inaccurately answered questions. Considering this and the fact that there were differences in the learning-relevant feedback-related ERPs between the growth and fixed mindset groups, the authors suggested that possibly there is greater attention allocation to learning-relevant feedback among growth-minded participants.

Even though the results from these neuroscientific studies focusing on mindsets are somewhat controversial and lack replication, they seem to still consistently refer to differences in the ERPs between growth- and fixed-minded individuals. It is important to take into consideration that almost all of the above-mentioned results have been found in a single study not yet having been replicated, which leaves them tentative and in need for additional confirmative findings. Moreover, exploration of feedback-related ERPs and their associations with mindsets have been especially rare and, as far as we know, have not previously been studied in children. Furthermore, academic domain specificity of mindsets has not yet been investigated in neuroscientific studies regarding implicit beliefs. The current study, which is part of the “Copernicus – Changing Mindsets about Learning: Connecting Psychological, Educational and Neuroscientific Evidence” project, aims to address this gap by examining general intelligence and academic-domain-specific, more specifically math ability mindsets, and their relations to automatic reactions to performance-relevant feedback in mathematics in the Finnish elementary school context. The academic domain of mathematics was chosen since achievement in mathematics is often believed to depend more on an uncontrollable innate ability when compared to achievement in other domains, for example, social sciences and languages (Gunderson et al., 2017; Costa and Faria, 2018). Additionally, students seem to consider mathematics to be one of the most important and difficult school subjects (Dundar et al., 2014). In the current study, elementary school students completed an age-appropriate math task that provided performance-relevant feedback throughout the task, while their ERPs and performance were recorded. We focused on exploring FRN and P300, which, as mentioned earlier, have been in the focus of neuroscientific research on reactions to feedback. FRN and P300 below refer to their difference amplitudes between negative and positive performance-relevant feedback in the math task.

Taking into account the findings from the previous studies described above, we expected to find:

1. no relationship between overall accuracy on the task and mindsets (both general intelligence and math ability), since no previous study found such a relationship (Mangels et al., 2006; Moser et al., 2011; Schroder et al., 2014, 2017);
2. no relationship between RTs and mindsets (both general intelligence and math ability), since no previous study found such a relationship (Moser et al., 2011; Schroder et al., 2014, 2017);
3. a stronger endorsement of growth mindset (both regarding general intelligence and math ability) to be related to higher PEA, since growth mindset has been associated with better self-regulatory processes in case of failure and behavioral adjustment after setbacks (Moser et al., 2011; Burnette et al., 2013);
4. the association between math ability mindset and PEA in the math task to be stronger than the one between general intelligence mindset and PEA, since academic-domain-specific beliefs predict outcomes in that specific academic domain better than general intelligence beliefs or mindsets regarding another domain (Gunderson et al., 2017; Costa and Faria, 2018);
5. no relationship between FRN and mindsets (both general intelligence and math ability), since significant associations with the negative deflection following errors or negative feedback have not been found (Mangels et al., 2006; Moser et al., 2011; Schroder et al., 2014, 2017);
6. mindsets (both regarding general intelligence and math ability) to be associated with the P300 amplitude, since mindsets have previously been shown to associate with feedback-related P300 amplitude (Mangels et al., 2006);
7. the association between math ability mindset and P300 in math task to be stronger than the one between general intelligence mindset and P300 in math task, since academic-domain-specific beliefs have been shown to predict outcomes in that specific academic domain better than the beliefs regarding general intelligence or another domain (Gunderson et al., 2017; Costa and Faria, 2018);
8. P300 amplitude to be associated with PEA, since the previous studies have shown P300 to be associated with attentional resources directed toward the stimulus (Polich, 2007) and to predict subsequent error correction (Butterfield and Mangels, 2003; Mangels et al., 2006; Ernst and Steinhauser, 2012).

## Materials and Methods

### Participants

The participants of our study were 97 third-grade students (46 girls, 46 boys, and 5 did not report their gender; *M*_age_ = 8.94 years, *SD*_age_ = 0.43) from two Finnish public elementary schools. Both schools are located in the Helsinki metropolitan area, one in a low socioeconomic status (SES) area and the other in a medium SES area (Vilkama et al., 2014).

### Materials

#### Mindset Measures

In order to measure the participants’ general intelligence mindset, an instrument including the four Entity Theory items from the Implicit Theories of Intelligence Scale (Dweck, 1999) was used. The original scale consists of four Entity Theory statements (e.g., *You have a certain amount of intelligence, and you cannot really do much to change it*) and four Incremental Theory statements (e.g., *You can always substantially change how intelligent you are*). Following Dweck’s recommendations, the latter ones were not included in the current questionnaire as these items are not reliable due to social desirability, and thus, using Entity Theory statements is a standard practice in this research area (Dweck, 1999). For measuring participants’ math ability mindset, the same four Entity Theory statements from the Implicit Theories of Intelligence Scale were adapted to be math ability specific. Participants indicated how much they agreed with each statement by marking one of the six circles that varied in size ranging from *not at all* to *really a lot*, which mapped to a 6-point Likert-type scale. Higher scores indicate a greater endorsement of growth mindset. The internal consistencies of the instruments were acceptable (general intelligence mindset Cronbach’s *ɑ* = 0.75; math ability mindset Cronbach’s *ɑ* = 0.79).

#### Math Task

**Figure 1:**
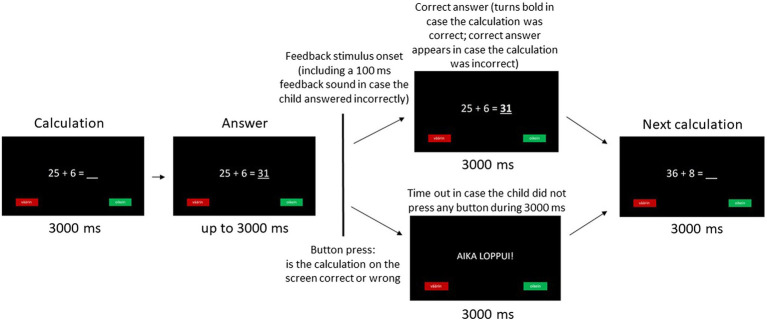
Math task.

The participants’ ERPs to feedback in mathematics were recorded during the completion of an age-appropriate math-specific two-alternative choice task (Figure 1). Each trial of the task consisted of a math calculation with one number missing from the calculation that was presented at a central location on the computer monitor for 3000 ms. After this, either a correct or wrong answer appeared in the place of the missing number at most for 3000 ms. During this 3000 ms response window, the participants were instructed to press one of the two buttons on a response box with their dominant hand in order to indicate whether they thought the number appearing in the calculation was the correct answer or not. The participant’s response was followed by the bolded correct answer on the monitor (in case of a correct equation on the screen) or by the incorrect answer changing to a correct one (in case of an originally incorrect equation on the screen) for 3000 ms. In case of an incorrect response, a feedback tone of 100 ms followed immediately in order to ensure that the participant was aware of having made a mistake. In case the participant did not press any button during the 3000 ms response window, a time-out message appeared in the center of the monitor for 3000 ms before the next trial. The task consisted of a practice block (5 correct equation trials and 5 incorrect equation trials) to ensure that the participants had understood the task. According to the participants’ performance during the practice block, they were subsequently administered an easier (0–5 trials answered correctly) or more difficult version (6–10 trials answered correctly) of the actual task in order to ensure that the calculations in the task would be challenging enough but not too difficult for the participants. The actual task consisted of two blocks (47 trials in the first block and 46 trials in the second block) making up a total of 93 trials. The 93 trial calculations (48 correct equations and 45 incorrect equations) were presented in a random order for each participant. The children were allowed a 5- to 10-min refreshment pause between the blocks. The positions of the two buttons on the response box were alternated every second experimental day in order to avoid possible motor response confounds in the aggregated data (Grootswagers et al., 2017).

### Procedure

The children’s participation in this study was voluntary, and parental, school principals’, municipal officials’ written consents were obtained. The children and their parents were informed about the study procedures and their right to cancel their participation at any moment of the study and measurements. The research project for the study was reviewed and approved beforehand by the University of Helsinki Ethical Review Board.

The questionnaire regarding general intelligence and math ability mindset was administered to the participants by a researcher as part of a longer questionnaire during their regular school hours. The researcher read each question and response options out loud as the participants correspondingly filled in the electronic questionnaire behind laptops or tablets provided by the school. The procedure lasted approximately for 40 min.

The experiment, including the math task and psychophysiological recording, was conducted by one to two experimenters in a separate room at the school premises during regular school hours. Before the experiment, the children were briefed about the process of the experiment and reminded of their right to cancel their participation at any moment. After completing the task, the children were compensated with sweets and stickers for their participation. The whole procedure lasted approximately 1 h and 15 min per participant.

### Data Recording and Processing

Continuous electroencephalographic activity was recorded with portable equipment (BrainVision QuickAmp amplifier) using 32 Ag-AgCl active electrodes (ActiCap, Brain Products, Germany). Electrolyte gel (Signa Gel, Bio-Medical Instruments, Inc., Warren, MI) was used at each electrode. The data were recorded with BrainVision Recorder at 250 Hz sampling rate. Recording reference was Fpz or FCz depending on the size of the used cap.

After recording, the EEG data were processed with MATLAB R2019a software (Mathworks, Natick, MA) with EEGLAB 19.0 toolbox. The signal was band-pass filtered with cutoffs of 0.1 Hz and 30 Hz and segmented into epochs beginning 200 ms before button press and continuing for 750 ms following button press. In addition to visual inspection, artifactual epochs were rejected by detecting abnormal trends and abnormal spectra, and eye movement artifacts were removed using independent component analysis (Delorme and Makeig, 2004). The data were subsequently re-referenced to the mean of the mastoid electrodes.

Feedback-locked ERPs were calculated relative to a −150 to −50 ms baseline window, which was also approximately −150 to −50 ms pre-response (button press) as the time difference between the button press and feedback stimulus onset was only a few milliseconds. In order to obtain feedback-related ERPs regarding participants’ authentic decisions about the accuracy of the math calculations and in order to exclude trials with accidental button presses, all trials where the RT was less than 300 ms post-stimulus (the answer appearing in the place of the missing number of the equation on the screen) were left out from the analyses (Thomas et al., 1981). Also, time-out trials were excluded from further analyses. Additionally, to ensure reliable averages of ERPs, a minimum of six trials was considered necessary for each participant for both error and correct trials in order to calculate the averages (Pontifex et al., 2010). The average number of correct trials included in the further analyses was 42 (min 20, max 71) and the number of error trials was 27 (min. 6, max 53) per participant. Subsequently, the averaged ERPs for correct trials were subtracted from the averaged ERPs for error trials and the aggregated amplitude curve was visually inspected in order to determine the time windows for ERPs to be quantified. Additionally, topographical maps from these time windows were created and visually inspected to determine electrode sites where ERPs were maximal. Accordingly, feedback-locked grand average ERPs for three electrode sites along the scalp midline (Fz, Cz, and Pz) were calculated.

**Figure 2:**
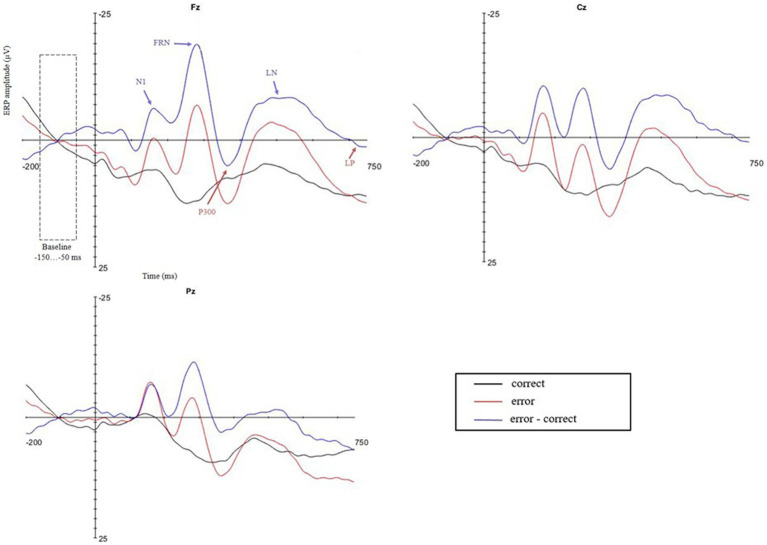
Feedback-locked waveforms for positive and negative feedback trials at frontal Fz, central Cz, and parietal Pz electrodes with indicated baseline and ERP time windows: N1, FRN, P300, LN, and LP. The 0 point on the time scale represents the feedback stimulus onset. Analyzed ERP amplitudes were collected based on individual peak latencies: FRN within the 200–360 ms, P300 within the 250–500 ms, LN within the 360–625 ms, and LP within the 500–725 ms time window after feedback stimulus onset.

**Figure 3:**
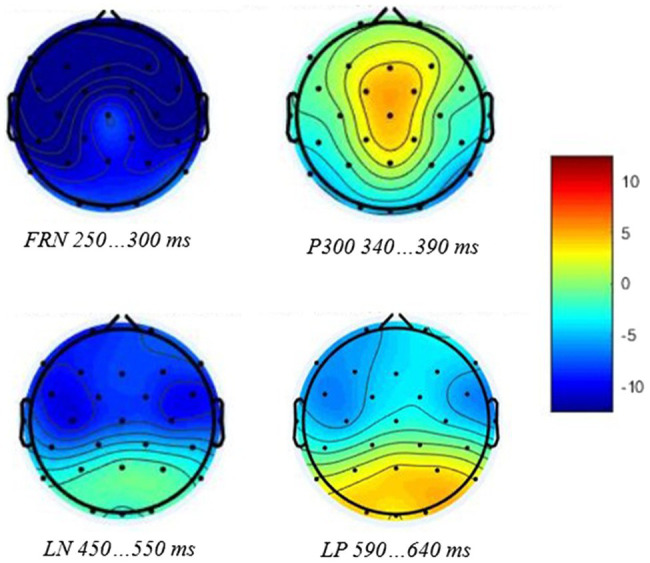
Scalp distribution maps for FRN and P300 (above), and LN and LP (bottom) amplitude differences between negative and positive feedback trials.

The first negative peak was observed at 50–200 ms after the onset of the feedback stimulus, and taking into account the experimental design of the study, it was presumably affected by the N1 response elicited by the negative feedback sound on error trials (Figure 2). Additionally, preliminary analyses showed no associations between this first negative peak and mindsets or behavioral data, and consequently, it was excluded from further analyses. A subsequent negatively displaced response, which peaked between 200 and 360 ms after feedback stimulus onset, was identified as FRN (Figures 2, 3). FRN was assessed as mean difference amplitude over 50 ms time window around each participant’s negative peak between latencies 200 and 360 ms. P300 was calculated as mean difference amplitude over 50 ms time window around each participant’s positive peak between latencies 250 and 500 ms after feedback stimulus onset. We also observed one later emerging negative deflection peaking between 360 and 625 ms after feedback stimulus onset and one later emerging positive deflection peaking between 500 and 725 ms after feedback stimulus onset. We termed the negatively displaced response as late negativity (LN) and the positively displaced response as late positivity (LP) due to their latencies. LN was assessed as mean difference amplitude over 100 ms time window around each participant’s negative peak between latencies 360 and 625 ms, and LP was calculated as mean difference amplitude over 50 ms time window around each participant’s positive peak between latencies 500 and 725 ms after feedback stimulus onset.

In order to estimate the consistency of these observed brain responses, split-half reliabilities using Spearman-Brown coefficient for each observed response at midline electrode sites for correct and error trials were computed (Hajcak et al., 2017). The first 14 correct and error trials were included for computing the internal reliabilities as including more has been shown to result in only slight enhancement in the reliability coefficient while losing subjects due to the lack of sufficient number of accepted trials (Hajcak et al., 2017). As some participants had less than 14 artifact-free trials, the number of trials included in the calculations for internal reliabilities was smaller than 14 in the case of these participants. All of the split-half reliability coefficients for each ERP component for correct and error trials at the three electrode sites were above 0.74, which indicates a sufficient reliability of these responses (all of the split-half reliability coefficients can be found in the Supplementary Table S1).

Behavioral measures from the math task included overall accuracy, RTs, post-error and post-correct RTs, and accuracy. PEA was calculated as sum of the number of correct answers following error trials divided by sum of number of all answers following error trials. Post-correct accuracy (PCA) was calculated, respectively, using the sum of number of correct answers following correct trials.

### Data Analyses

First, descriptive statistics of mindset, behavioral and ERP variables were calculated (Tables 1, 2), and the normality of data distribution was visually inspected. As the variables were normally distributed, Pearson correlation was used to examine the relationships between the study variables (correlations can be found in the Supplementary Table S2). Subsequently, the data were checked to ensure that other assumptions for general linear modeling in addition to normality were satisfied. In case the assumption of sphericity was not satisfied, Greenhouse-Geisser correction was used. After this, repeated-measures analyses of variance (rANOVAs) were conducted on behavioral measures in order to check for the differences between error and correct trials, and subsequently, the scores of general intelligence mindset (GEN) and math ability mindset (MATH) were entered into the rANOVAs as continuous predictors to explore the main effects of mindsets and interactions between mindsets and behavioral measures.

**Table 1:** Descriptive statistics of questionnaire and behavioral variables.

Variable	*M* (*SD*)	Minimum, Maximum
General intelligence mindset	3.66 (1.19)	1.00, 6.00
Math ability mindset	4.16 (1.20)	1.25, 6.00
Overall accuracy (%)	60.7 (10.8)	38.1, 88.8
PEA (%)	60.3 (12.2)	30.0, 94.1
PCA (%)	61.1 (11.3)	38.5, 92.0
RT (ms)	1701 (286)	983, 2189
EH RT (ms)	1767 (307)	934, 2275
CH RT (ms)	1675 (288)	1035, 2229
Post-EH CH RT (ms)	1713 (312)	1029, 2278
Post-CH CH RT (ms)	1651 (295)	1015, 2325
Post-error slowing (%)	104.4 (13.0)	64.9, 143.5

EH, error hit; CH, correct hit.

**Table 2:** ERP components: paired-samples *t*-test results along with descriptive statistics for mean error trial, correct trial, difference amplitudes, and peak latencies.

ERP components	Electrode site	Error trial *M* (*SD*) in μV	Correct trial *M* (*SD*) in μV	Paired-samples *t*-test		Error minus correct	
				*t*(96)	*p*	Amplitude *M* (*SD*) in μV	Peak latency *M* (*SD*) in ms
FRN	Fz	−6.29 (19.41)	11.21 (19.07)	−14.90	0.000	−17.81 (11.55)	277 (20)
	Cz	0.74 (18.58)	10.81 (18.58)	−8.99	0.000	−9.99 (9.94)	279 (24)
	Pz	−4.11 (16.67)	6.72 (18.02)	−9.77	0.000	−7.21 (8.92)	280 (27)
P300	Fz	12.70 (20.62)	7.70 (21.29)	3.81	0.000	6.23 (12.64)	373 (42)
	Cz	16.70 (19.59)	10.14 (19.89)	4.79	0.000	7.93 (12.97)	365 (43)
	Pz	13.17 (16.71)	9.04 (19.27)	3.51	0.001	6.22 (12.41)	393 (49)
LN	Fz	−5.79 (20.61)	4.82 (23.43)	−7.82	0.000	−11.97 (13.49)	526 (70)
	Cz	−2.91 (20.31)	7.34 (22.14)	−7.53	0.000	−11.85 (13.26)	513 (71)
	Pz	1.67 (19.14)	5.58 (20.83)	−2.91	0.004	−6.02 (12.72)	500 (79)
LP	Fz	10.37 (26.77)	10.15 (24.13)	0.12	0.906	3.03 (18.47)	643 (77)
	Cz	12.49 (25.14)	11.10 (22.73)	0.80	0.429	3.56 (17.40)	643 (72)
	Pz	16.03 (24.95)	7.39 (21.30)	4.45	0.000	9.49 (18.79)	641 (70)

Regarding ERPs, in order to first check for the differences between error and correct trials, paired-samples *t*-tests were conducted to examine whether the error and correct trial ERP amplitudes were significantly different from each other. Subsequently, rANOVAs or, when appropriate, univariate ANOVAs (UNIANOVAs) were conducted on ERP measures, including GEN and MATH scores as continuous predictors in order to assess the main effects of mindsets and interactions between mindsets and responses. In order to explore the relationships between ERP responses and post-error behavioral measures, rANOVAs or, when appropriate, UNIANOVAs on ERP measures, including PEA as continuous predictor, were conducted. In case of significant effects, follow-up analyses were conducted to aid with the interpretation of the results.

## Results

### Mindsets

As expected, a wide range of mindset endorsements was observed with most participants’ mindset scores falling between fixed and growth extremes (Table 1). Next, the relationship between GEN and MATH was examined. A significant, intermediate correlation between GEN and MATH was observed (*r* = 0.41, *p* < 0.01).

### Behavioral Data

The descriptive statistics of behavioral data from the two-choice task are presented in Table 1. On average, the participants were correct on 60.7% (*SD* = 10.8%) of the trials (excluding time-out trials) with the average accuracy on the completion of the easier version of the task (*N* = 37) being 57.7% (*SD* = 8.8%) and the accuracy for the more difficult version (*N* = 60) being 62.6% (*SD* = 11.5%). Regarding Hypothesis 1, the overall accuracy was not related to mindsets in either of the difficulty levels of the task (*p* > 0.09). RTs on error trials (*M* = 1767 ms) were significantly longer than RTs on correct trials (*M* = 1675 ms) [*F*(1,96) = 24.05, *p* < 0.001, *η*^2^ = 0.20]. Concerning Hypothesis 2, when mindsets were entered into the rANOVA as continuous predictors, there were no significant effects (all rANOVA results can be found in the Supplementary Table S3).

Regarding post-error behavioral data, post-error RTs on subsequent correct trials (*M* = 1713 ms) were significantly longer than post-correct RTs on following correct trials (*M* = 1651 ms) [*F*(1,96) = 8.59, *p* = 0.004, *η*^2^ = 0.08], indicating a PES effect. When mindsets were entered into the rANOVA as continuous predictors, there were no significant effects (Supplementary Table S3). There was no significant difference between PEA (*M* = 60.3%) and PCA (*M* = 61.1%). Regarding Hypotheses 3 and 4, there were no significant effects when mindsets were entered into the rANOVA as continuous predictors (Supplementary Table S3).

### Feedback-Related ERPs

#### Feedback-Related Negativity

Feedback-related negativity was the second negative deflection after the N1 (Figure 2). According to the paired-samples *t*-test, error and correct trial FRN amplitudes differed significantly from each other at all three electrode sites, indicating a significant difference between error trial and correct trial responses (Table 2). In order to test Hypothesis 5, FRN was then analyzed using rANOVA, including FRN difference amplitudes from three electrode sites (Fz, Cz, and Pz) with GEN and MATH as continuous predictors. The main effect of GEN was not significant, and neither was the interaction between GEN and electrode site (Supplementary Table S3). The main effect of MATH was not significant, and neither was the interaction between MATH and electrode site (Supplementary Table S3). However, a main effect of electrode site emerged [*F*(1.67,156.48) = 5.10, *p* = 0.01, *η*^2^ = 0.05], with post-hocs indicating that FRN was larger at Fz than Cz or Pz electrodes (*p* < 0.001).

#### P300

According to the paired-samples *t*-test, error and correct trial P300 amplitudes differed significantly from each other at all three electrode sites, indicating a significant difference between error trial and correct trial responses (Table 2). In order to test Hypotheses 6 and 7, P300 was then analyzed using rANOVA, including P300 difference amplitudes from three electrode sites (Fz, Cz, and Pz) with GEN and MATH added as continuous predictors. The main effect of GEN was not significant, and neither was the interaction between GEN and electrode site (Supplementary Table S3). The main effect of MATH approached significance [*F*(1,94) = 3.49, *p* = 0.07, *η*^2^ = 0.04], indicating that higher growth mindset regarding math ability was marginally associated with larger P300 difference amplitude [with lower quartile scores (3.5) P300: mean at *Fz* = 5.01 μV, mean at *Cz* = 6.58 μV, mean at *Pz* = 4.79 μV; with higher quartile scores (5.25) P300: mean at *Fz* = 8.24 μV, mean at *Cz* = 10.14 μV, mean at *Pz* = 8.56 μV]. The interaction between MATH and electrode site was not significant (Supplementary Table S3). Additionally, no significant main effect of the electrode site emerged, indicating that there was no difference in the P300 at the three electrode sites (Supplementary Table S3).

#### Late Negativity

According to the paired-samples *t*-test, error and correct trial LN amplitudes differed significantly from each other at all three electrode sites, indicating a significant difference between error trial and correct trial responses (Table 2). LN was then analyzed using rANOVA, including LN difference amplitudes from three electrode sites (Fz, Cz, and Pz) with GEN and MATH as continuous predictors. The main effect of GEN was not significant, and neither was the interaction between GEN and electrode site (Supplementary Table S3). The main effect of MATH was significant [*F*(1,94) = 4.61, *p* = 0.03, *η*^2^ = 0.05], indicating that higher growth mindset regarding math ability was associated with smaller LN difference amplitude [with lower quartile scores (3.5) LN: mean at *Fz* = −13.43 μV, mean at *Cz* = −13.61 μV, mean at *Pz* = −7.68 μV; with higher quartile scores (5.25) LN: mean at *Fz* = −9.57 μV, mean at *Cz* = −8.98 μV, mean at *Pz* = −3.24 μV]. The interaction between MATH and electrode site was not significant (Supplementary Table S3). Additionally, no significant main effect of the electrode site emerged, indicating that there was no difference in the LN at the three electrode sites (Supplementary Table S3).

#### Late Positivity

According to the paired-samples *t*-test, error and correct trial LP amplitudes differed significantly from each other only at Pz electrode site, indicating a significant difference between error trial and correct trial responses only at the parietal site (Table 2). LP was then analyzed using UNIANOVA, including LP difference amplitude from the parietal electrode site with GEN and MATH added as continuous predictors. The effects of GEN and MATH were not significant (Supplementary Table S3).

### Brain-Behavior Relationships

In order to examine brain-behavior relationships, rANOVA on FRN was conducted with PEA added as a continuous predictor. There was no significant main effect (Supplementary Table S3), but a significant interaction between PEA and electrode site emerged [*F*(1.61,153.28) = 4.61, *p* = 0.02, *η*^2^ = 0.05], indicating that PEA was differently associated with FRN at different electrode sites. However, subsequent separate UNIANOVA analyses for each electrode revealed that these associations were not significant [*F*(1,95) ≤ 3.17, *p* ≥ 0.08, *η*^2^ ≤ 0.03].

Subsequently, in order to test Hypothesis 8, rANOVA on P300 with PEA added as a continuous predictor was conducted. There was no significant main effect (Supplementary Table S3), but a significant interaction between PEA and electrode site emerged [*F*(1.62,153.42) = 4.53, *p* = 0.02, *η*^2^ = 0.05], indicating that PEA was differently associated with P300 amplitudes at different electrode sites. However, subsequent separate UNIANOVA analyses for each electrode revealed that these associations were not significant [*F*(1,95) ≤ 2.03, *p* ≥ 0.16, *η*^2^ ≤ 0.02].

Next, rANOVA on LN with PEA added as a continuous predictor was conducted. There was neither significant main effect nor interaction between PEA and electrode site (Supplementary Table S3).

Finally, UNIANOVA on LP with PEA added as a continuous predictor was conducted. The main effect of PEA was significant [*F*(1,95) = 11.37, *p* = 0.001, *η*^2^ = 0.11], indicating that higher PEA was associated with larger LP amplitude at the parietal electrode site [with lower quartile scores of PEA (52%) LP amplitude mean at *Pz* = 5.31 μV; with higher quartile scores of PEA (68%) LP amplitude mean at *Pz* = 13.34 μV].

## Discussion

The neuroscientific research on mindsets, especially among children, is still scarce, and none of the previous studies in this field has taken academic domain specificity of mindsets into account. We aimed to address this gap, and thus in the current study, we examined the relations of general intelligence and academic-domain-specific, more specifically math ability mindsets to automatic reactions to negative feedback in mathematics in Finnish elementary school students. We found P300, the positive deflection thought to index attention processes related to working memory, to be marginally associated with mindsets and LN, a later peaking negatively displaced response, to be significantly associated with mindsets, while for FRN, the negative deflection reflecting initial detection of outcome valence, and for LP, a positive-going waveform with later latency, no such association was found. More specifically, we found that a larger P300 amplitude and a smaller LN amplitude elicited by negative feedback in math were associated with higher growth mindset regarding math ability (in the case of P300 this association being only marginal), but not with mindset regarding general intelligence. As associations between academic-domain-specific mindsets and ERPs elicited by feedback in the corresponding domain had previously not been explored, the results of this study offer new insight for understanding the complexity and specificity of mindsets in action.

### Mindsets

The moderate positive correlation between general intelligence and math ability mindset suggests that these mindsets are related, but still separable from one another, which is consistent with the previous research. Namely, it has been suggested that there are a general factor and domain-specific facets to mindsets (Dweck et al., 1995; Schroder et al., 2016).

### Behavioral Data

Confirming our expectations in Hypothesis 1, the overall accuracy in the math task was not related to mindsets. This is consistent with the previous studies (Mangels et al., 2006; Moser et al., 2011; Schroder et al., 2014, 2017). Longer RTs on error trials, when compared to RTs on correct trials, are inconsistent with the results of the previous studies using a speeded reaction time task (Moser et al., 2011; Schroder et al., 2014, 2017). This is probably due to the differences between the tasks used in the previous studies and the current one. Unlike the previous research, we did not employ a simple speeded-response task, but required the participant to calculate prior to their response instead of simply reacting to the stimulus as fast as possible. The longer RT on error trials in our study could indicate that it was more demanding for the participants to calculate their answers on those trials or that they were more hesitant regarding their answers on error trials. Confirming Hypothesis 2 and consistently with earlier studies, mindsets did not have any significant effects on RTs (Moser et al., 2011; Schroder et al., 2014, 2017).

Regarding post-error behavioral data, the post-error RTs on the following correct trials were significantly longer than the post-correct RTs on the following correct trials, indicating a PES effect, which is consistent with the previous studies using a speeded reaction-time error-monitoring task (Moser et al., 2011; Schroder et al., 2014, 2017). Again, mindsets did not have any significant effects on post-error RTs, which is also in line with the previous studies (Moser et al., 2011; Schroder et al., 2014, 2017).

Consistently with the previous studies, there was no difference between PEA and PCA (Moser et al., 2011; Schroder et al., 2014, 2017). Inconsistently with our expectations in Hypotheses 3 and 4, mindsets had no significant effects on PEA, which is compatible with one previous study (Schroder et al., 2014) but inconsistent with others, where higher growth mindset was either marginally (Schroder et al., 2017) or significantly associated with higher PEA relative to PCA (Moser et al., 2011).

Thus, consistently with earlier research, we did not find associations between behavioral data and mindsets, but inconsistently with some earlier studies, we found no association between mindsets and PEA, either. According to the mindset theory, for someone with a fixed mindset, a failure or making a mistake rather refers to the lack of their natural ability needed to succeed, as opposed to seeing it as an indication of the need to imply more effort or a different strategy (Molden and Dweck, 2006). This can subsequently lead fixed-minded individuals to avoid challenges and give up when facing failure (Molden and Dweck, 2006). Theoretically, it could be expected for a higher growth mindset to be associated with higher PEA as the growth-minded person would see an error and the performance-relevant feedback in this case as a sign of the need to implement more effort and focus on the following trials. Nevertheless, this was not the case, which possibly suggests that the task used in the current study demanded more than simply applying more effort or focus in order to succeed as it was not a regular speeded reaction time task, but a more demanding and complex math calculation task. Additionally, the previous research has also shown that learning goals and effort attributions mediate the relationship between growth mindset and adaptive post-failure behavior without a direct significant effect between the mindset and behavior (Smiley et al., 2016). Thus, it could also be speculated that in the case of a more complex task, as the one used in this study, the participating growth-minded children did not attribute their mistakes simply to their lack of effort.

### Feedback-Related ERPs

#### Feedback-Related Negativity

We observed a negatively displaced FRN response with maximal amplitude difference at Fz following negative feedback, peaking between 200 and 360 ms after feedback stimulus onset. This frontally maximal negative deflection following negative feedback is compatible with earlier research on performance-relevant feedback-related ERPs (Miltner et al., 1997; Butterfield and Mangels, 2003; Mangels et al., 2006). Regarding Hypothesis 5 concerning the relationship with mindsets, there were no significant associations between FRN and mindsets, which is compatible with the previous research (Mangels et al., 2006). The study by Mangels et al. (2006) is, as far as we know, the only earlier study focusing on associations between mindsets and feedback-related ERPs, while most of the neuroscientific research on mindsets has examined error-related ERPs in speeded reaction time tasks (Moser et al., 2011; Schroder et al., 2014, 2017). These studies explored ERN, the negative-going waveform following the commission of errors, and found no relationship between mindsets and this negative deflection associated with initial error detection (Moser et al., 2011; Schroder et al., 2014, 2017). Earlier research on error- and feedback-related ERPs and corresponding equivalent dipole analysis has suggested that FRN appears to reflect the same neural process as ERN (Miltner et al., 1997). Thus, consistently with the previous research, our results suggest that mindsets are not related to the initial detection of the outcome valence itself.

#### P300

In addition to FRN, we observed P300, a positive deflection peaking between 250 and 500 ms after the onset of the feedback stimulus. This positive deflection following feedback is compatible with the previous research on feedback-related ERPs (Butterfield and Mangels, 2003; Mangels et al., 2006; for review, see Glazer et al., 2018). P300 amplitude did not differ between the midline recording sites, which might be due to its more frontal P3a and more parietal P3b subcomponents overlapping (Polich, 2007). Regarding Hypothesis 6 concerning the associations with mindsets, the P300 amplitude was only marginally associated with mindsets. Earlier research exploring the relationships between mindsets and feedback-related ERPs found a greater frontally maximal P300, possibly reflecting the P3a subcomponent, to be associated with fixed mindset and endorsement of performance goals (Mangels et al., 2006). This association was thought to indicate the greater salience of the negative performance feedback among fixed-minded participants. Interestingly, in our study, the direction of this association, though not reaching statistical significance, indicated a larger P300 amplitude to be associated with higher growth mindset. Hence, our marginally significant result does not comply with the findings of Mangels et al. (2006). It is important to mention, though, that in the study by Mangels et al. (2006), this frontally maximal P300 response was elicited by performance-relevant feedback stimulus, but in our study, performance-relevant feedback was presented simultaneously with corrective feedback. Thus, in this case, a larger P300 could indicate more attentional resources engaged in the processing of the corrective feedback stimulus. Complying with this speculation, Schroder et al. (2014) found larger P300 to incongruent trials among the participants in the growth mindset induction group when compared to the fixed mindset induction group. These results could indicate greater attention allocation to stimulus processing after growth mindset induction. Additionally, error-related ERP studies have found higher growth mindset to be associated with a larger Pe response elicited by errors in a speeded reaction time task (Moser et al., 2011; Schroder et al., 2017). These results have been interpreted as growth-minded individuals allocating more attention to errors with Pe mediating the effect of growth mindset on post-error adjustment (Moser et al., 2011). Thus, taking into account the findings of Schroder et al. (2014) and that Pe and P300 have been suggested to reflect similar processes involved in conscious processing of motivationally significant events (Ridderinkhof et al., 2009), the results of the present study regarding the amplitude of P300 seem to comply with these previous findings.

Additionally, regarding Hypothesis 7, the domain-specific experimental design of the current study provided informative findings concerning the academic domain specificity of mindsets. Namely, a larger P300 amplitude elicited by negative feedback in math was marginally associated with higher growth mindset regarding math ability, but the association between the P300 amplitude and mindset regarding general intelligence did not approach significance. Even though these findings only approached statistical significance, it could possibly refer to the importance of not only domain but also academic domain specificity of mindsets (Gunderson et al., 2017; Costa and Faria, 2018).

#### Late Negativity

In addition to the FRN and P300, we observed a negative-going waveform following the P300 response and peaking between 360 and 625 ms after feedback stimulus onset. Regarding the topographical distribution of this response, the LN amplitudes did not differ at the midline electrode sites. Such a late negative-going waveform, as far as we know, has not previously been reported in feedback-related ERP studies. Interestingly, in our study, this LN amplitude was associated with mindsets. Namely, higher growth mindset in math ability was associated with a smaller LN difference amplitude elicited by feedback in the math task. It is important to highlight that the effect size for this association was small, indicating that math ability mindset only explains a very small percentage of the variance in the amplitudes of the LN response. Nevertheless, this significant association, although small in effect size, was observed only in the case of mindsets regarding math ability. Namely, general intelligence mindset had no association, not even a marginal one, with the LN amplitude during the math task. When examining the latencies of P300 and LN observed in the current study and taking into account the later peaking and longer lasting character of the P3b subcomponent of the P300 canonical waveform, it could be speculated that the positive-going P3b, associated with memory processes, could be overlapping with the subsequent negative-going LN response. In this case, a smaller LN difference amplitude could possibly reflect a greater latent P3b difference amplitude. As we found a greater P300 difference amplitude to be marginally associated with a growth mindset in math ability, the significant association with a smaller LN amplitude could possibly reflect the underlying association between growth mindset in math ability and a greater latent P3b difference amplitude. Nevertheless, these results are novel and as such a LN elicited by feedback has not been observed in the previous studies, this association remains to be explored by future research.

#### Late Positivity

The other late deflection following performance feedback was a positive-going waveform emerging at the parietal site after the LN response and peaking between 500 and 725 ms after feedback stimulus onset. This type of a later emerging positive waveform has not previously been reported in feedback-related ERP research focusing on mindsets (Mangels et al., 2006). A later sustained positive-going centro-parietal ERP beginning at around 500–600 ms and possibly continuing for several seconds after stimulus onset has been examined in the context of reward processing assumed to reflect sustained attention toward and elaborative processing of emotionally and motivationally salient stimuli (Weinberg and Hajcak, 2011; Pornpattananangkul and Nusslock, 2015; for review, see Glazer et al., 2018). It could be speculated that this late positive-going waveform observed in the current study could reflect sustained attention to and further processing of the feedback stimulus. Regarding the relationship with mindsets, though, there were no significant associations observed with the LP response. Thus, it remains unclear, which processes this later emerging positive waveform reflects in the context of feedback processing.

### Brain-Behavior Relationships

PEA did not have a significant main effect regarding FRN, which is consistent with the suggestion that FRN codes outcome valence and that the need for behavioral adjustment is not its core feature (Von Borries et al., 2013). Not complying with our expectations in Hypothesis 8, PEA did not have a significant association with the P300 amplitude. This is contradictory to earlier findings that found corrective feedback-related P300 to be larger for initial errors that were answered correctly in the subsequent retest (Butterfield and Mangels, 2003; Mangels et al., 2006; Ernst and Steinhauser, 2012). In the present study, though, the corrective and performance-relevant feedback were presented simultaneously; thus, the P300 amplitude in the current study reflects attention not only toward the learning-relevant stimulus, but also toward the performance-relevant stimulus. Additionally, in the present study, behavioral adjustment was not measured using a retest enabling the assessment of the later accuracy of initial errors, but simple PEA. Thus, instead of reflecting the attentional resources directed at the specific learning-relevant stimulus, higher PEA in this design could rather reflect general heightened attention toward the overall task following errors and the accompanying feedback.

There were no associations between PEA and LN. Regarding the positive-going LP, though, PEA had a significant effect. Namely, higher PEA was associated with larger LP at the parietal site. This suggests that the observed LP could reflect heightened and sustained attention on the task following errors. A later emerging positive deflection following negative feedback has been linked to subsequent behavioral adjustment also in earlier studies (San Martín et al., 2013; Von Borries et al., 2013; for review see Glazer et al., 2018). Thus, the found association between LP and PEA seems to support the assumption of LP reflecting attention to motivationally salient stimuli coupled with subsequent behavioral adjustment (Glazer et al., 2018).

### Limitations

As our study explored only general intelligence mindsets and mindsets about a single academic domain – math – regarding the reactions while completing a math-specific task, it has limitations that should be addressed in the future. To make more reliable conclusions regarding academic domain specificity of mindsets in action, the experimental design should compare several different academic-domain-specific mindsets, for example, math ability and writing ability mindsets, and their relations to automatic reactions to feedback in math-specific and writing-specific tasks. Another option could be including an additional task, performance on which would be associated with general intelligence. Such a design would enable comparing general intelligence and math ability mindsets, and their relations to automatic reactions to feedback in general intelligence and math-specific tasks. Also, the inclusion of a feedback sound in case of an inaccurate response is a considerable limitation of the current study, making it more challenging to compare positive and negative feedback-related ERPs. Yet, in our study, we prioritized to study the reactions to feedback that would be clear and could not be perceived as ambiguous by the participants. Thus, the decision to use the feedback sound was made to make the participants clearly aware of their errors and the valence of the feedback.

Additionally, the design of the current study limits the exploration of the performance-relevant feedback-related ERPs separately from the ERPs related to corrective learning-relevant feedback. This limits the interpretation of the results of the current study. In the future, performance-relevant and corrective feedback could be presented separately in order to be able to differentiate between the ERPs elicited by performance-relevant feedback stimulus and learning-relevant feedback stimulus.

Another limitation to address concerns the mindset measures, which were self-report questionnaires. Using self-report questionnaires among this age group might be problematic regarding understanding of the questions and self-reflection necessary to answer them (Borgers et al., 2000). In the future, the assessments of teachers and parents could additionally be used regarding mindset measures.

### Conclusion

To conclude, our results suggest that mindsets about math ability might be linked to attentional processing of the feedback received regarding performance in the domain of math. These results suggest that domain specificity of mindsets might matter when it comes to the complex interaction of implicit beliefs and feedback in the process of interpretation and meaning making by the student. Namely, mindsets regarding specific domains possibly play a bigger role in eliciting automatic reactions to feedback in the corresponding domains when compared to more general mindsets. Moreover, even though earlier research has shown domain-specific and general mindsets to have a general factor in addition to domain-specific aspects, our results regarding automatic reactions to feedback suggest that it might be important to address domain-specific and even academic-domain-specific beliefs in addition to general mindsets when planning interventions and looking for ways to support students’ learning. Nevertheless, these observed changes in ERP amplitudes associated with mindsets in the current study were not associated with subsequent behavioral adjustment and the changes in ERP amplitudes associated with improved subsequent performance were not associated with mindsets. Thus, even though the results regarding the observed automatic reactions suggest that domain specificity of mindsets could matter in the process of meaning making and interpretation by the student, the ways in which these beliefs and their interactions with processing feedback get translated into behavioral outcomes are not so straightforward. Thus, these math ability- and other academic-domain-specific mindsets and their role in students’ behavioral outcomes in the corresponding academic domains call for further research.

## Data Availability Statement

The raw data supporting the conclusions of this article will be made available by the researchers of the Copernicus project, without undue reservation.

## Ethics Statement

The studies involving human participants were reviewed and approved by University of Helsinki Ethical Review Board in the Humanities and Social and Behavioral Sciences. Written informed consent to participate in this study was provided by the participants’ legal guardian/next of kin.

## Author Contributions

IP, MHuo, TK, SL, EK, and KT planned the experimental design. IP, MHuu, and KK collected and pre-processed the data. IP and TL conducted the analyses. IP, TL, MHuu, KK, MHuo, TK, SL, EK, and KT wrote the paper. All authors contributed to the article and approved the submitted version.

## Conflict of Interest

The authors declare that the research was conducted in the absence of any commercial or financial relationships that could be construed as a potential conflict of interest.

## Publisher’s Note

All claims expressed in this article are solely those of the authors and do not necessarily represent those of their affiliated organizations, or those of the publisher, the editors and the reviewers. Any product that may be evaluated in this article, or claim that may be made by its manufacturer, is not guaranteed or endorsed by the publisher.

## Abbreviations

ERN: error-related negativity
ERP: event-related potential
FRN: feedback error-related negativity
GEN: general intelligence mindset
LN: late negativity
LP: late positivity
MATH: math ability mindset
Pe: error positivity
PCA: post-correct accuracy
PEA: post-error accuracy
RT: reaction time
