# Supplementary material for: Mindsets and Neural Mechanisms of Automatic Reactions to Negative Feedback in Mathematics in Elementary School Students
Source: Front Psychol. 2021 Aug 6;12:635972. doi: 10.3389/fpsyg.2021.635972 (PMC8377164; doi:10.3389/fpsyg.2021.635972)
Supplement: Supplementary file 1 [file Table_1.docx]

Supplementary Material

# Table S1

*Split-half reliabilities of each ERP response at midline electrode sites using*

*Spearman-Brown coefficient (including first 14 correct and error trials)*

| ERP variable | Electrode site | Spearman-Brown coefficient | |
| --- | --- | --- | --- |
|  |  | correct trial | error trial |
| FRN | Fz | .85 | .78 |
|  | Cz | .85 | .74 |
|  | Pz | .83 | .78 |
| P300 | Fz | .86 | .79 |
|  | Cz | .83 | .81 |
|  | Pz | .84 | .74 |
| LN | Fz | .87 | .79 |
|  | Cz | .86 | .78 |
|  | Pz | .87 | .79 |
| LP | Fz | .81 | .81 |
|  | Cz | .80 | .80 |
|  | Pz | .81 | .77 |

**Table S2**

*Pearson correlations among variables. Only significant responses have been included in the table.*

| Variable | 1 | 2 | 3 | 4 | 5 | 6 | 7 | 8 | 9 | 10 | 11 | 12 | 13 | 14 | 15 | 16 | 17 | 18 | 19 | 20 |
| --- | --- | --- | --- | --- | --- | --- | --- | --- | --- | --- | --- | --- | --- | --- | --- | --- | --- | --- | --- | --- |
| 1. GEN |  |  |  |  |  |  |  |  |  |  |  |  |  |  |  |  |  |  |  |  |
| 2. MATH | .41** |  |  |  |  |  |  |  |  |  |  |  |  |  |  |  |  |  |  |  |
| 3. Overall accuracy (%) | .00 | .10 |  |  |  |  |  |  |  |  |  |  |  |  |  |  |  |  |  |  |
| 4. PEA (%) | -.02 | .08 | .90** |  |  |  |  |  |  |  |  |  |  |  |  |  |  |  |  |  |
| 5. PCA (%) | -.02 | .11 | .91** | .67** |  |  |  |  |  |  |  |  |  |  |  |  |  |  |  |  |
| 6. RT (ms) | -.04 | .01 | .07 | .04 | .11 |  |  |  |  |  |  |  |  |  |  |  |  |  |  |  |
| 7. EH RT (ms) | -.06 | .02 | .33** | .26* | .35** | .90** |  |  |  |  |  |  |  |  |  |  |  |  |  |  |
| 8. CH RT (ms) | -.05 | .02 | .00 | -.02 | .06 | .98** | .81** |  |  |  |  |  |  |  |  |  |  |  |  |  |
| 9. Post-EH CH RT (ms) | -.05 | .04 | .06 | .01 | .12 | .94** | .83** | .93** |  |  |  |  |  |  |  |  |  |  |  |  |
| 10. Post-CH CH RT (ms) | -.03 | .00 | -.02 | -.03 | .03 | .90** | .71** | .94** | .77** |  |  |  |  |  |  |  |  |  |  |  |
| 11. Post-error slowing (%) | -.06 | .06 | .13 | .08 | .13 | .10 | .22* | .02 | .38** | -.30** |  |  |  |  |  |  |  |  |  |  |
| 12. FRN at Fz | -.01 | .08 | .21* | .10 | .22* | -.05 | -.02 | -.04 | -.08 | .01 | -.11 |  |  |  |  |  |  |  |  |  |
| 13. FRN at Cz | -.05 | .03 | .29** | .18 | .31* | .01 | .06 | .01 | -.02 | .05 | -.09 | .78** |  |  |  |  |  |  |  |  |
| 14. FRN at Pz | .07 | .08 | .10 | -.02 | .14 | -.04 | -.02 | -.05 | -.07 | -.03 | -.05 | .68** | .85** |  |  |  |  |  |  |  |
| 15. P300 at Fz | -.01 | .14 | .03 | -.02 | .03 | -.16 | -.13 | -.17 | -.15 | -.18 | .06 | .54** | .51** | .52** |  |  |  |  |  |  |
| 16. P300 at Cz | .07 | .18 | .26* | .15 | .27** | -.08 | -.02 | -.10 | -.06 | -.12 | .11 | .50** | .63** | .60** | .82** |  |  |  |  |  |
| 17. P300 at Pz | .16 | .24* | .11 | -.03 | .14 | .00 | .03 | -.01 | .01 | -.04 | .09 | .37** | .53** | .66** | .67** | .83** |  |  |  |  |
| 18. LN at Fz | -.05 | .14 | .05 | .04 | .02 | -.03 | -.03 | -.05 | -.04 | -.07 | .09 | .29** | .26* | .34** | .60** | .48** | .58** |  |  |  |
| 19. LN at Cz | -.04 | .18 | .09 | .09 | .04 | -.02 | -.02 | -.03 | -.01 | -.06 | .12 | .21* | .28** | .35** | .49** | .50** | .63** | .89** |  |  |
| 20. LN at Pz | .00 | .20 | .09 | .03 | .09 | .01 | .02 | .00 | -.01 | -.03 | .05 | .16 | .26** | .43** | .41** | .48** | .73** | .76** | .88** |  |
| 21. LP at Pz | .00 | .16 | .35** | .33** | .32** | -.10 | .04 | -.13 | -.11 | -.15 | .07 | .03 | .08 | .13 | .13 | .21* | .29** | .48** | .56** | .60** |

*Note:* EH = error hit; CH = correct hit. * indicates *p* < .05. ** indicates *p* < .01.

# Table S3

*rANOVA results*

| Dependent variable | Predictor | *df_Num_* | *df_Den_* | *F* | *p* | *η2* |
| --- | --- | --- | --- | --- | --- | --- |
| EH RT vs CH RT |  | 1 | 96 | 24.05 | **0.00** | 0.20 |
|  | GEN | 1 | 94 | 0.48 | 0.49 | 0.01 |
|  | MATH | 1 | 94 | 0.23 | 0.63 | 0.00 |
| Post-EH CH RT vs Post-CH CH RT |  | 1 | 96 | 8.59 | **0.00** | 0.08 |
|  | GEN | 1 | 94 | 0.33 | 0.57 | 0.00 |
|  | MATH | 1 | 94 | 0.19 | 0.66 | 0.00 |
| PEA vs PCA |  | 1 | 96 | 0.54 | 0.46 | 0.01 |
|  | GEN | 1 | 94 | 0.47 | 0.50 | 0.01 |
|  | MATH | 1 | 94 | 1.44 | 0.23 | 0.02 |
| N1 |  |  |  |  |  |  |
|  | GEN | 1 | 94 | 0.01 | 0.95 | 0.00 |
|  | MATH | 1 | 94 | 0.40 | 0.53 | 0.00 |
| FRN |  |  |  |  |  |  |
|  | Electrode Site | 1.67 | 156.48 | 5.10 | **0.01** | 0.05 |
|  | GEN | 1 | 94 | 0.08 | 0.78 | 0.00 |
|  | GEN x Electrode Site | 1.67 | 156.48 | 1.33 | 0.27 | 0.01 |
|  | MATH | 1 | 94 | 0.50 | 0.48 | 0.01 |
|  | MATH x Electrode Site | 1.67 | 156.48 | 0.27 | 0.72 | 0.00 |
|  | PEA | 1 | 95 | 0.89 | 0.35 | 0.01 |
|  | PEA x Electrode Site | 1.61 | 153.28 | 4.61 | **0.02** | 0.05 |
| P300 |  |  |  |  |  |  |
|  | Electrode Site | 1.72 | 161.91 | 2.49 | 0.09 | 0.03 |
|  | GEN | 1 | 94 | 0.01 | 0.93 | 0.00 |
|  | GEN x Electrode Site | 1.72 | 161.91 | 2.26 | 0.12 | 0.02 |
|  | MATH | 1 | 94 | 3.49 | 0.07 | 0.04 |
|  | MATH x Electrode Site | 1.72 | 161.91 | 0.08 | 0.90 | 0.00 |
|  | PEA | 1 | 95 | 0.11 | 0.74 | 0.00 |
|  | PEA x Electrode Site | 1.62 | 153.42 | 4.53 | **0.02** | 0.05 |
| LN |  |  |  |  |  |  |
|  | Electrode Site | 1.58 | 153.95 | 1.11 | 0.32 | 0.01 |
|  | GEN | 1 | 94 | 1.34 | 0.25 | 0.01 |
|  | GEN x Electrode Site | 1.58 | 153.95 | 0.30 | 0.69 | 0.00 |
|  | MATH | 1 | 94 | 4.61 | **0.03** | 0.05 |
|  | MATH x Electrode Site | 1.58 | 153.95 | 0.21 | 0.76 | 0.00 |
|  | PEA | 1 | 95 | 0.30 | 0.58 | 0.00 |
|  | PEA x Electrode Site | 1.57 | 149.15 | 0.61 | 0.50 | 0.01 |
| LP* |  |  |  |  |  |  |
|  | GEN | 1 | 94 | 0.47 | 0.49 | 0.01 |
|  | MATH | 1 | 94 | 2.90 | 0.09 | 0.03 |
|  | PEA | 1 | 95 | 11.37 | **0.00** | 0.11 |

*Note.* EH = error hit; CH = correct hit. *df_Num_* indicates degrees of freedom numerator. *df_Den_* indicates degrees of freedom denominator. Significant p-values are marked in bold.

*Due to only one electrode showing significant response, the analysis was conducted with UNIANOVA.
